# Early removal of the etonogestrel contraceptive implant in Spanish women: a prospective cohort study

**DOI:** 10.3389/fmed.2024.1172793

**Published:** 2024-01-23

**Authors:** Regina Ruiz de Viñaspre-Hernández, Rosana Garrido-Santamaria, Raquel Urra-Martínez, Paula Sáenz-Cabredo, Ana Elena Garrido-Rivas, Raúl Juárez-Vela, Juan Luis Sánchez-González, Alberto Lafuente-Jimenez, Enrique Ramón-Arbués, Noelia Navas-Echazarreta, Ivan Santolalla-Arnedo

**Affiliations:** ^1^Faculty of Health Sciences, University of La Rioja, Logroño, Spain; ^2^Grupac Research Group, Department of Nursing, University of La Rioja, Logroño, Spain; ^3^Family Planning Center, Rioja Health Service, Logroño, Spain; ^4^Rioja Health Service, Logroño, Spain; ^5^Department of Nursing and Physiotherapy, University of Salamanca, Salamanca, Spain; ^6^Faculty of Health Sciences, San Jorge University, Villanueva de Gállego, Spain; ^7^SAPIENF Investigation Group, University of Zaragoza, Zaragoza, Spain

**Keywords:** etonogestrel, contraceptive, implant, early removal, cohort study

## Abstract

**Purpose:**

To know the discontinuation rate and characterize predictors and reasons of contraceptive implant removal within 12 months of insertion in our community setting.

**Methods:**

This prospective cohort study included women receiving the etonogestrel contraceptive implant at sexual and reproductive health centers between September 2019 and September 2020. The variables collected were implanted insertion timing, reproductive and demographic characteristics, medical conditions, sexual activity and counseling. Our primary outcome was implant discontinuation. Kaplan–Meier survival curves were used to show the cumulative discontinuation rate of implants contraceptive within the first year of insertion. We also identified factors that increased the risk of implant removal using the log-rank test and the Cox regression model. Reasons for discontinuation were documented.

**Results:**

199 women were followed up. Implant discontinuation was documented in 17.1% of implant users prior to 12 months. Factors that increase the risk of implant removal are living with a partner, being aged 25–34 years and not receiving comprehensive and structured counseling from the midwife. The main reason for removal was unsatisfactory bleeding (97.1%), but this was combined with other reasons such as cessation of sexual intercourse (58.8%), worsening mood (58.8%), weight gain (55.9%) or decreased libido (50.0%).

**Conclusion:**

The rate of discontinuous implant uses in the first year is relevant in relation to cost-effectiveness, there is room for improvement that should not be overlooked. Comprehensive and structured midwife-led counseling can reduce early implant abandonment removal. The development in different countries of the role of midwives in the management of contraceptives can contribute to the economic benefit of health services and the satisfaction of women.

## Introduction

Women’s sexual and reproductive health (SRH) is associated with access to highly effective contraceptive methods that minimize the risk of having to bear the physical and psychological consequences of unwanted childbearing or abortion. The unintended pregnancy reduces maternal and infant quality of life, educational attainment, job opportunities and economic stability, and increases the economic costs for health system Worldwide, it is estimated that avoiding teenage pregnancies, spacing pregnancies, and ending unwanted pregnancies and abortions could prevent one in three maternal deaths (1). Furthermore, to exercise the right to sexual health and well-being, it is essential that women be able to choose their sexual practices without fear of pregnancy (2).

Etonogestrel subdermal implant (ESI) is a long-acting reversible contraceptive (LARC) which is safe and highly effective (3). Its effect is based on the slow and sustained release of etonogestrel which induces anovulation, inadequate development of the endometrium and thickening of the cervical mucus. In Spain it is marketed as Implanon NXT, an implant with a single, soft, flexible plastic rod containing 68 milligrams of the active ingredient, etonogestrel, and is currently approved by the European Medicines Agency for 3 years of use.

Studies report wide variability in continuation rates at one year after placement. The Moray 2021 review showed rates of 57 to 97%, and the two meta-analyses conducted in the same review showed that pooled one-year continuation rate was 77.5 and 76.5%, respectively. Continuation rates were higher in lower-middle-income or low-income countries compared to high-income countries (4). European studies also show this variability, with continuation rates of 72% (5) or 91% (6).

ESI commonly causes changes in the frequency, intensity, or duration of vaginal bleeding. In the first three months, it is estimated that irregular bleeding appears in 50% of users and prolonged bleeding in 30% (7) and these bleedings are the most frequent reason for early implant removal (8). Other reasons are headache, weight gain, changes in mood, decreased libido, skin conditions (acne, hair loss, or hirsutism), dizziness, lower abdominal pain, mastalgia, fatigue or localized pain at the insertion site, among others (9).

In Spain, implant insertion is free of charge and the health-care system funds between 40 and 100% of the pharmaceutical cost, depending on the woman’s income. Despite its high efficacy and the few medical situations that discourage its use (10), only 1.4% of Spanish women who use contraceptives had an implant in 2019 (11). Both low acceptance and early removal of the implant have been associated with non-existent or inadequate contraceptive counseling (8, 12).

Implant placement requires an initial financial outlay that is highly cost-effective for both the woman and the health system that funds it, provided it is not removed too early. In a weighted cost analysis in women aged 20–29, cost neutrality was estimated to be achieved at 2.1 years (13). Therefore, when looking at women’s interests and the sustainability of the health system, it is necessary to provide data on early discontinuation and the reasons that best explain it, aiming to establish measures that prevent early removal and result in greater cost savings. The objective of our study is to understand the discontinuation rate in the first year of implant use, as well as the conditions of the women that have an early implant removal and the reasons that they have for withdrawing the implant.

## Methods

### Prospective cohort study

The study started in September 2019 at the Centre for Sexual and Reproductive Health (CSHR) in La Rioja (Spain), data collection for this study was performed during one year (2019–2020).

### Context of the study

La Rioja is a territory located in the north of Spain with an area of 5,000 km^2^ and a population of 319,485 inhabitants, of which 47% live in the city of Logroño (150,020 inhabitants), 30% in towns of between 25,000 and 5,000 inhabitants and 23% in towns of less than 5,000 inhabitants. It has a network of 19 health centers distributed throughout its geography and the community midwife is part of the primary care team in all of them. Travel to the city does not exceed 45 min.

In 2019, the protocol of care for women requesting contraception included a comprehensive and standardized contraceptive counseling program that is modeled on the structure used in the CHOICE Project (14) whose components provide accurate and unbiased information to help women assess their needs and make an informed decision, within the framework of the Shared Decision Making (15). This type of structured contraceptive counseling service was led by community midwives, who had been previously trained. In a face-to-face visit lasting 15 to 20 min, oral and written information was provided, and doubts were resolved for an informed and shared decision on contraception. From this consultation, the midwives arranged a quick appointment at the CSHR, the only health care center where trained personnel (midwife or gynecologist) fitted the implants. Follow-up visits were scheduled at 3, 6, and 12 months and they were offered the possibility of making an appointment whenever they needed. Other women went to the CSHR on their own accord, without first attending the midwife’s consultation. These women received the routine counseling usually provided at the CSHR prior to implant insertion. Three months after implant insertion, a review appointment is scheduled at the community midwife’s office or at the same sexual and reproductive health center. Access to the community midwife’s consultation and the CSHR was universal, free and direct. The women paid between 0 and 60 euros per implant, and the health system paid the rest of the amount.

### Participants

All women of childbearing age who, between 09/09/2019 and 09/08/2020, went to the CSHR for implant placement were invited to participate. The exclusion criteria were: lack of cognitive or language ability to understand and sign the consent for participation and the refusal of the legal representative or guardian of a girl under 16 years old to sign the informed consent. All eligible women agreed to participate.

The study was approved by the ethics committee of La Rioja (Ref. CEImLAR P.I 386).

The participants underwent a standardized contraceptive evaluation in the family planning protocol to identify medical situations that contraindicated its use (10). The research staff collected the data reported by the patient in relation to her socio-demographic status (age, place of residence, nationality, studies, work relationship, affective relationship, cohabitation with a partner), gynecological and obstetric history (pregnancies, births, abortions, living children, history of STIs, previous use of hormonal contraceptives), medical conditions (BMI, smoking and physical exercise), sexual activity (sexual satisfaction and frequency of intercourse) and counseling. After pregnancy was ruled out, the implant was inserted by a qualified professional. Follow-up visit was scheduled at 3 month.

The dependent variable -discontinuation rate- was defined as the cumulative probability that women who received the implant have it removed in the first year. The independent variables were the sociodemographic and medical variables collected in the contraceptive evaluation: age was categorized into three ranges (15 to 24 years old, 25 to 34 years old and 35 or more years old); place of residence (capital city with 150,022 inhabitants, towns of 25,000 to 5,000 inhabitants and towns with less than 5,000); stability of the relationship: stable (little tension, little uncertainty, low-risk break-up) or unstable (tension, uncertainty, high-risk break-up); cohabitation as a couple: yes (usually sharing the same household) or no (usually living in different houses) partner relationship: monogamous (a couple) and non-monogamous (several couple and single): yes or no; employment situation: unemployed, salaried employee, self-employed, student; education level: compulsory education was defined as period of education that is required of all people and is imposed by the Spanish government (up to 16 years old) or non-compulsory (above 16 years old); country of birth: Spain (native women) or other country (migrant women), physical exercise was defined as the performance of some activity in order to develop or maintain physical fitness and overall health, 1 or more days a week; yes or no; smoker yes or no; Body Mass Index (BMI) was categorized into four groups: underweight (BMI <20 kg/m2), normal weight (20–24.9 kg/m2), overweight (25–29.9 kg/m2) and obese class I (≥30 kg/m2); frequency of sexual intercourse: occasional, monthly, weekly or daily; history of hormonal contraceptives and Sexually Transmitted Infections (STIs): yes or no; history of pregnancy, childbirth, voluntary abortion: yes or no and having children: yes or no. Sexual satisfaction was defined as overall contentment with emotional and sexual aspects of the sex life and was assessed with the validated Spanish version of the sexual satisfaction scale for women (SSS-W-e), at the beginning of the method. The scale that measures contentment is made up of 6 items: 1. I feel content with my present sex life; 2. I feel some-thing is missing from my present sex life; 3. I feel I do not have enough emotional closeness; 4. I feel content with the frequency of sexual intimacy; 5. I do not have any problems or concerns about sex; 6. Overall, I am satisfied with my sex life. Women are instructed to rate their level of agreement with each item using a Likert scale from 1 = strongly disagree to 5 = strongly agree. The score range is 6–30, and it is calculated by adding the scores of the individual items (16). Sexual satisfaction was low when its value was below the median and high when it was above the median. Type of counseling: Women were considered to have received “midwife-led structured counseling” when they had attended the scheduled consultation with the community midwife. Women who did not use this service were provided with the “usual counseling.”

Women who requested implant removal in the first year were asked to complete an *ad-hoc* questionnaire, on the day of implant removal, to collect two types of information: (a) their level of satisfaction, with the safety, comfort and price of the method and the accessibility of the service (ease and speed of appointment for implant insertion and removal) on a scale of 0 to 10 (from zero to maximum satisfaction), and (b) the reason(s) leading them to request implant removal: the questionnaire included a predetermined list and they could indicate only one or more than one, together with an open question to add other reasons not contemplated in the previous list. Both questionnaires were designed based on a literature review and information collected in the medical records of other women who had their implants removed before the start of the study.

### Statistical analysis

Women’s characteristics and reasons for early implant removal were reported in terms of frequency and percentage and satisfaction levels with median and interquartile range. To estimate the discontinuation rate, we will calculate the cumulative incidence based on the Kaplan–Meier approach (exact-events times) and to assess whether there are differences in the distribution of method discontinuation times in the first year, based on different factors, we will calculate the chi-square using the log-rank test. The Cox regression model was used to estimate the risk of discontinuing contraception in the first year. Variables that showed statistical significance in the log-rank test were included in the model and those that continued to show statistical significance in the model were retained (*p* < 0.05).

## Results

### Sample description

**Figure 1:**
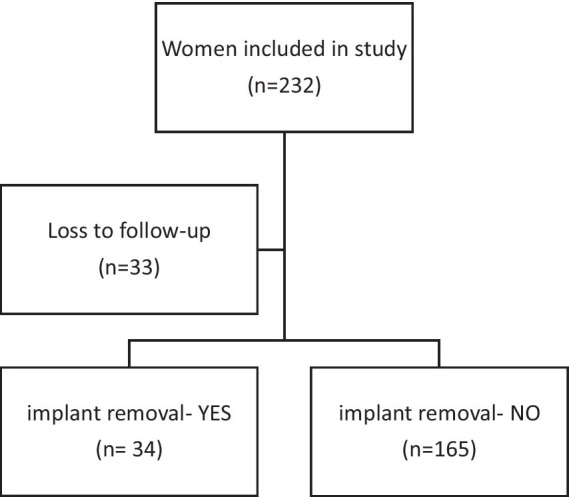
Flow diagram of cohort.

232 women were recruited for the study, of whom 33 (14.2%) could not be traced in the first year of follow-up. The remaining 199 (85.8%) continued in the study. Of these 199 women, 34 (17.1%) had the implant removed within the first year (Figure 1).

The observed cohort (199 women) was between 15 and 50 years old. The majority were migrant women,117 (60.3%) in monogamous 166 (83.8%) and stable160 (80.8%) relationships and lived with their partner 124 (62.6%). Almost 117 (60%) were employed while the other 80 (40%) were unemployed 49 (25%) or students 31 (15%), and 86 (45%) of the women had compulsory education (up to the age of 16). Among them, 83 (41.75%) were of normal weight, 77 (39.1%) exercised at least once a week, the majority 127 (64.5%) did not smoke. For 71 (57.7%) of the women their sexual satisfaction was high and most of them had sex at least once a week 109 (56.5%). Approximately half of the women had received prior advice from the midwife (Table 1).

**Table 1:** Baseline women characteristics.

Variables	All women (*N* = 199)		Removal implant (*N* = 34)	
	*n*	%	*n*	%
Age (group)				
15–24 years old	84	42.2	9	26.5
25–34 years old	72	36.2	20	58.8
35 or more years old	43	21.6	5	14.7
Place of residence				
Capital city (150,022 inhabitants)	106	53.3	17	50.0
Towns (25,000–5,000 inhabitants)	64	32.2	13	38.2
Towns (< 5,000 inhabitants)	29	14.6	4	11.8
Stability of the relationship				
Unstable (high-risk break-up)	38	19.2	3	8.8
Stable (low-risk break-up)	160	80.8	31	91.2
Sexual Relationship (partner)				
Monogamous (a couple)	166	83.8	30	88.2
Non-monogamous* (several couple)	20	10.1	3	8.8
Non-monogamous* (single)	12	6.1	1	2.9
Cohabitation as a couple				
No	74	37.2	7	20.6
Yes	124	62.6	27	79.4
Employment situation				
Unemployed	49	24.9	10	29.4
Salaried employee	109	55.3	19	55.9
Self-employed	8	4.1	2	5.9
Student	31	15.7	3	8.8
Education level				
Non-compulsory (above 16 years)	105	55.0	18	52.9
Compulsory (up to 16 years old)	86	45.0	16	47.1
Country of birth				
Spain (native women)	77	39.7	13	38.2
Other country (migrant women)	117	60.3	21	61.8
Physical exercise				
No	120	60.9	19	57.6
Yes	77	39.1	14	42.4
Smoker				
No	127	64.5	22	66.7
Yes	70	35.5	11	33.3
Body mass index (BMI) category**				
Low weight (BMI <20 kg/m^2^)	22	11.1	5	14.7
Normal weight (BMI = 20–24.9 kg/m^2^)	83	41.7	11	32.4
Overweight (BMI = 25–29.9 kg/m^2^)	59	29.6	10	29.4
Obesity (BMI ≥30 kg/m^2^)	35	17.6	8	23.5
Sexual intercourse (frequency)				
Occasional	24	12.4	2	5.9
Monthly	39	20.2	10	29.4
Weekly	109	56.5	19	55.9
Daily	21	10.9	3	8.8
History of hormonal contraceptives				
No	66	38.2	6	18.8
Yes	123	61.8	26	81.3
History of STIs^#^				
No	171	88.1	27	84.4
Yes	23	11.9	5	15.6
Type of counseling				
Midwife-led structured counseling	86	49.1	21	70
Usual counseling	89	50.9	9	30
History of pregnancies				
No	54	27.1	6	17.6
Yes	145	72.9	28	82.4
History of childbirth				
No	78	39.2	9	26.5
Yes	121	60.8	25	73.5
History of voluntary abortions				
No	129	65.2	22	64.7
Yes	69	34.8	12	35.3
Children^##^				
No	76	38.2	9	26.5
Yes	123	61.8	25	73.5
Sexual satisfaction^###^				
Low	52	42.3	11	55
High	71	57.7	9	45

*n* = frequency; % = percentage; *Non-monogamous = term for every practice of non-dyadic intimate relationship; **BMI = Body mass index (BMI) is defined as the body mass divided by the square of the body height and is expressed in units of kg/m^2^; ^**#**^STI, Sexually transmitted infections; ^##^Children: the woman has her own or adopted; ^**###**^Sexual satisfaction: low **=** values of the sexual satisfaction scale between the first and second quartiles and High = values between the third and fourth quartiles.

When comparing the socio-demographic and medical characteristics of the untraceable women to the women who remained in the study, we only found statistically significant differences (*p* > 0.05) in 4 of the 18 variables measured. Among the untraceable women there was a significantly higher proportion of Spanish women (χ^2^ = 4.388; *p* = 0.04), in non-stable relationships (χ^2^ = 4.715; *p* = 0.03), who did not live with their partners (χ^2^ = 4.043; *p* = 0.04), who were not monogamous (χ^2^ = 6.770; *p* = 0.03), nor did they practiced sport or physical exercise (χ^2^ = 5,671; *p* = 0.02).

**Figure 2:**
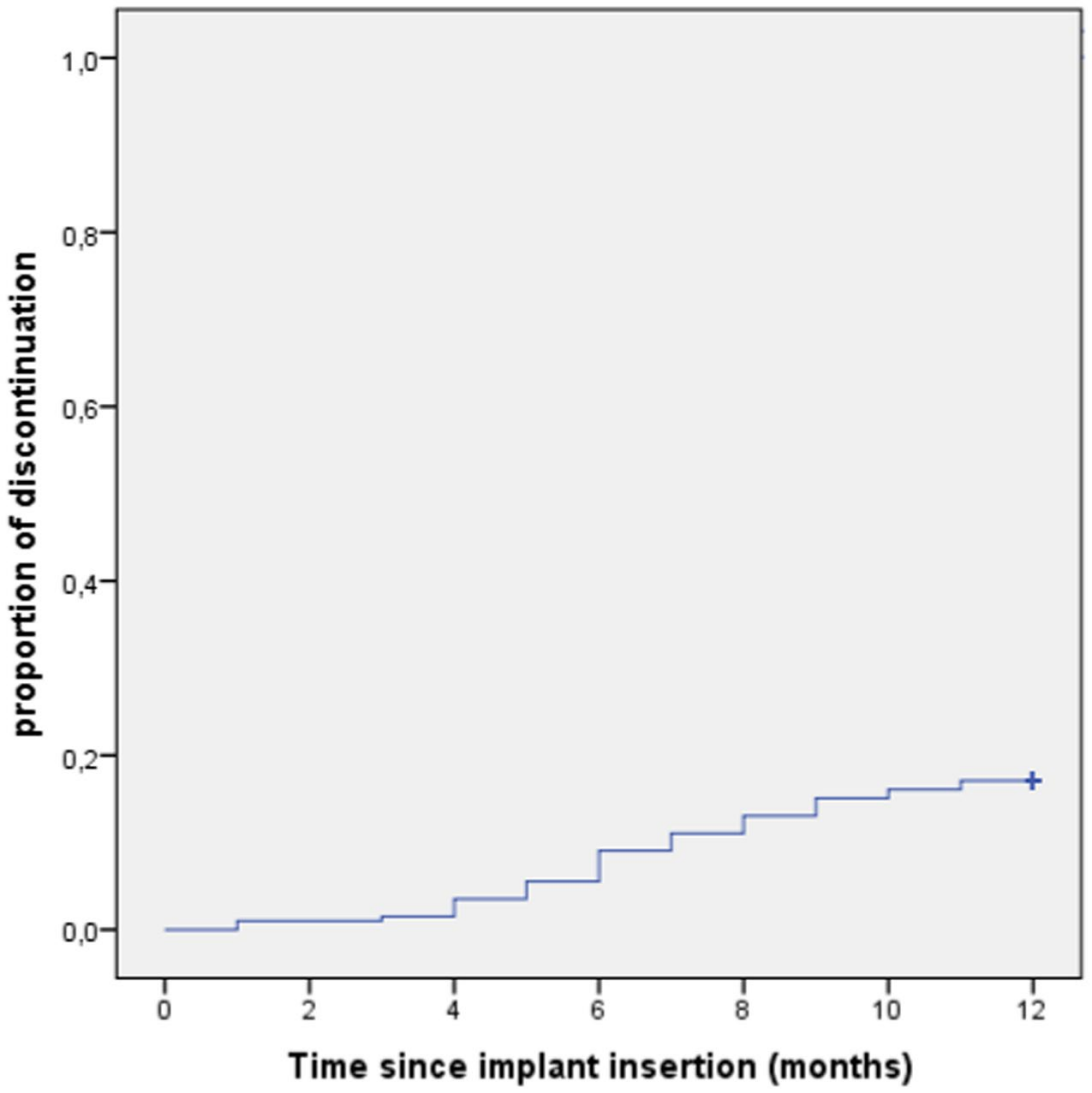
Survival analysis of first year discontinuation (Kaplan–Meier’s plots).

Discontinuation rate in the first 12 months was 17.1%. The mean of time to discontinuation (time form the start of implant to the end of implant) during the first year was 11.06 months (standard deviation 0.16) (Figure 2).

Table 2 shows that four variables were significantly linked to discontinuation time with the implant (age, cohabitation with partner, contraceptive counseling, and previous use of hormonal contraception). Of these, the first three remained statistically significant in the regression model (Table 3).

**Table 2:** Variables related to the time to discontinuation (TTD) with the implant.

Variables	Months of use		Log Rank (mantel-Cox)	
	Mean	SD	Chi-square	*p*-Value
Age				
15 y 24 years old	11.499	0.179	9.403	0.009
25–34 years old	10.417	0.339		
35 or more years old	11.302	0.320		
Place of residence				
Capital (150,022 inhabitants)	11.160	0.186	0.812	0.671
Towns of 25,000 to 5,000 inhabitants	10.965	0.312		
Towns less of 5,000 inhabitants	11.120	0.459		
Stability of the relationship				
Unstable	11.684	0.175	2.856	0.091
Stable	10.906	0.197		
Sexual relationship (partner)				
A couple	10.982	0.189	0.832	0.660
Several couple	11.167	0.460		
Single	11.667	0.319		
Cohabitation as a couple				
No	11.662	0.137	5.249	0.022
Yes	10.694	0.244		
Employment situation				
Unemployed	10.708	0.418	1.916	0.590
Salaried employee	11.028	0213		
Self-employed	10.875	0.926		
Student	11.633	0.247		
Education				
Non-compulsory	10.965	0.260	0.073	0.787
Compulsory	11.067	0.224		
Country of birth				
Spain	11.043	0.207	0.022	0.881
Other country	11.026	0.281		
Physical exercise				
No	11.136	0.203	0.217	0.642
Yes	10.908	0.288		
Smoker				
No	10.969	0.218	0.110	0.740
Yes	11.229	0.242		
Body mass index (BMI)				
Low weight (BMI <20)	10.818	0.533	2.235	0.522
Normal weight (BMI = 20–24.9)	11.205	0.243		
Overweight (BMI = 25–29.9)	11.237	0.245		
Obesity (BMI ≥30)	10.571	0.492		
Sexual intercourse (frequency)				
Occasional	11.708	0.200	3.479	0.323
Monthly	10.513	0.456		
Weekly	10.963	0.232		
Daily	11.571	0.327		
History of hormonal contraception				
No	11.576	0.179	4.131	0.042
Yes	10.803	0.233		
History of STI				
No	11.135	0.167	0.617	0.432
Yes	10.609	0.647		
Type of counseling				
Midwife-led structured counseling	11.337	0.2249	54.962	0.026
Usual counseling	10.831	0.226		
Pregnancies				
No	11.463	0.229	1.877	0.171
Yes	10.910	0.206		
Deliveries				
No	11.423	0.196	2.942	0.093
Yes	10.8126	0.235		
Voluntary abortions				
No	10.930	0.227	0.006	0.937
Yes	10.595	0.207		
Children				
No	11.434	0.191	2.478	0.115
Yes	10.829	0.235		
Sexual satisfaction				
Low	11.038	0.298	1.423	0.233
High	11.296	0.235		

Time to discontinuation, Time from the start of implant to the end of implant. SD = standard deviation; STI = Sexually transmitted infections; low sexual satisfaction = values of the sexual satisfaction scale between the first and second quartiles and High = values between the third and fourth quartiles.

**Table 3:** Proportional Hazards model (Cox’s regression).

Variable	Harz Ratio	95%CI	*p*-value
Cohabitation as a couple	3.174	(1.116–9.023)	0.030
Usual counseling*	3.520	(1.520–8.133)	0.003
Age**			0.044
15–24 years old	0.387	(0.150–0.999)	0.050
35 years or more	0.318	(0.115–0.879)	0.027

CI, confidence interval; *Women were not provided midwifery-led structured counseling; **Reference group: 25–34 years old.

In the first year after implant insertion, women who live with their partner are 3.4 times more likely to have the implant removed; if they do not receive midwife counseling, the risk is 3.4 times higher. Regarding age, younger women (aged 15–24 years) and older women (>35 years) have a lower risk of removal, 61 and 68% respectively, than women aged 25–34 years.

Satisfaction with the safety and comfort of the method, and accessibility rated between 8 and 10 points on a scale of 0 to 10. No woman became pregnant with the implant in the first year. The price of the implant was the least satisfactory aspect (Table 4).

**Table 4:** Evaluation of the women’ satisfaction with implant.

Degree of satisfaction with:	Median	IR
Security	10	1
Comfort	8	2
Price	6	4
Accessibility*	8.5	4

IR, Interquartile range. *Time delay from the woman’s decision to the insertion of the implant.

Only one woman reported a single reason for abandoning the method, and that was the desire to become pregnant in the short term. The rest of them, lists two or more reasons, the most common one (97.1%) was an excessive or unexpected bleeding pattern. However, in all cases, bleeding was accompanied by other reasons reported as such: 58.8% cessation of intercourse, 58.8% worsening of their mood, 55.9% weight gain, or 50% decreased libido (Table 5).

**Table 5:** Reasons given by women for removal the implant during the first year.

Reasons	Frequency	Percentage
Bleeding model changes (heavy o unpredictable bleeding)	33	97.1%
cessation of sexual relations	20	58.8%
mood disturbance (feelings of distress, nervousness, sadness or symptoms of depression, and anxiety)	20	58.8%
Weight gain	19	55.9%
Decreased livid	17	50.0%
Headaches	14	41.2%
Fluid retention or feeling of feeling of bloating.	14	41.2%
Skin changes (blemishes, acne, oiliness, hirsutism)	12	36.4%
Gastrointestinal disorders (vomiting, nausea, heartburn)	11	32.4%
Hair loss	9	26.5%
Breast pain	9	27.5%
Pain (colicky, abdominal, hypogastric, dysmenorrhea)	6	17.5%
Fatigue	4	12.5%
Desire for pregnancy	4	11.8%
Weight loss	2	5.8%
Dizziness	2	5.8%
Insertion arm pain	1	2.9%
Vaginal dryness	1	2.9%
Anemia	1	2.9%
Change of sexual orientation	1	2.9%
Pruritus vulvae	1	2.9%
cystitis	1	2.9%
Vaginal yeast infection	1	2.9%

## Discussion

Our study found that the rate of discontinuity with the implant one year after insertion was 17.1% and the conditions that increase the risk of removal were: living with an intimate partner, being between 25–34 years old and not attending the midwife for contraceptive counseling. Excessive or unexpected bleeding was a reason almost always present, but not enough on its own for the woman to decide to have the implant removed. The price of the implant was the worst rated aspect by women.

The characteristics of women who opted for the implant (40% of Spanish women and 60% of migrant women) do not correspond to the profile of women of childbearing age in La Rioja in 2019, where only 17.7% were migrant women. This finding was not an objective of our study, but we believe it is important to highlight. Another Spanish study already found that migrant women had a much higher use of LARC than Spanish women (17). We believe that this phenomenon could be associated with the persistence of misconceptions among Spanish clinicians that could lead to information biases (18). A paternalistic attitude in contraceptive counseling would lead to preferentially recommending the use of the implant to women with low economic resources or to non-compliant women (17, 19). The study by Loder (20) found that women with a higher perception of discrimination were more likely to use a highly effective, reversible method. It is possible that in our country’s health care model we have not managed to fully integrate person-centered care and that joint decision-making is not yet the norm when we talk about sexual and reproductive health. It is necessary that health professionals believe in women’s autonomy and train in those communication and counseling skills that make it possible (12).

.The discontinuation rate found in this study (17.1%) is similar to that reported in the national survey of the Spanish Society of Contraception in 2020 (16%) (11) and virtually identical to the rate reported by the CHOICE project (17%) in a cohort of 5,097 women in St. Louis, USA (21) or to the 16.7% found in another study in the Spanish population (22), albeit below the 9% also found in the Spanish population (6). Other European studies find somewhat lower continuation rates, in the Netherlands 26% (5) and in a multicenter clinical trial in Australia, Finland, Norway, Sweden and the United Kingdom 26.8% (9).

Among the factors that increase the risk of removal, counseling is the most easily rectifiable situation. We measured community midwife-led counseling because it was a programmed, homogenous intervention based on the structured contraceptive counseling provided by the Contraceptive CHOICE Project (14). This model, which includes counseling about the side effects of the method, has shown its effect on the choice and continued use of the implant (23) and subsequent studies support this result (12, 14). The parts of counseling that show greater efficiency are personalization of counseling and shared decision-making, questions about the patient’s reproductive life plan/pregnancy intentions and a discussion of contraceptive methods by level of effectiveness (24). Furthermore, the women who received prior advice from the community midwife had the opportunity to establish a relationship of trust that allowed her to return to visit her to resolve their doubts or complaints, which could avoid, in some cases, early removal of the implant.

According to our data, the risk of early implant removal is lower in women younger than 25 and older than 35. The review by Hendrick (2020) (25) included nine studies that considered age as a factor in the continuation of the LARC method (IUD and implant), the results were not consistent, one of the studies reported that the older the age at implant placement the higher the risk of early removal (26). Data from the CHOICE Project find no significant difference in continuation rates in relation to age (27).

A woman’s motivation to avoid pregnancy is associated with continuation of contraceptive use (28). A more precarious economic situation, for younger women, or an already satisfied reproductive project, for older women, may justify a stronger and irrevocable decision to avoid pregnancy. On the other hand, most Spanish women make their decision to become pregnant while living with their partner, a situation that facilitates access to housing, the sharing of expenses and childcare responsibilities, and therefore places them in a better position to take on an unplanned pregnancy (29). We believe that the conscious and unconscious cost/benefit balance that women make to continue with the implant could explain why some of them feel less motivated to avoid pregnancy and consequently may have a lower tolerance for the side effects of the method. In the CHOICE study, the only socio-demographic characteristic associated with removal before six months of LARC methods (IUD and implant) was being single (23). In Spain, the percentage of single women who live with their partners and have children is very significant. In 2019, according to data from the Spanish statistics institute (INE) the proportion of children born to unmarried mothers was 48.41%.

Excessive or unexpected bleeding is the main reason for early implant removal, which is consistent with the scientific literature (3, 4). The other reasons found have also been widely cited in the literature (8). The collected reasons are women’s perceptions, unproven but sufficient for them to decide to remove the implant. Changes in bleeding pattern is well-documented in the scientific literature (30, 31), however, there is no consistent evidence of the effect of the implant on weight gain (32) on mood (33, 34) or libido and sexual function (35), and it has even been found that perceived weight gain may be inaccurate and poorly correlated with actual weight (36). The mood disturbance and cessation of sexual intercourse was the seconds most frequently cited reasons for implant removal, but as in all cases this reason was accompanied by unsatisfactory bleeding; we cannot discriminate which of these reasons was more important in the woman’s decision. Other studies report a single reason for implant removal (8, 9), but most of our behaviors do not occur for a single reason, so it is necessary to understand the full range of women’s motivations and needs for choosing and continuing with a method (37). Open and ongoing communication between midwives and women could improve method adherence and women’s satisfaction (38).

### Implication for practice

Our data show the relevance of all women receiving counseling before implant insertion, that the counseling is structured and has demonstrated its effectiveness, and that health providers (midwives, nurses, and doctors) receive specific training to perform it. Informing women of the discomforts or side effects that may be associated with the implant allows them to decide in advance whether to accept them or not, improving their tolerance and adherence to the method. In addition, women can be provided with resources that mitigate these drawbacks, for example, strategies to improve libido, avoid vaginal dryness, weight changes, or fluid retention. Regarding changes in the bleeding pattern, only an excessive increase in bleeding could put the woman’s health at risk. In this case, it is suggested to rule out underlying pathology, provide drugs that relieve anemia and fatigue or pain if they appear and maintain close contact until checking how the bleeding evolves and how it affects the woman.

### Strengths and limitations

This study included all women who chose the implant for contraception for one year, no woman refused to participate, which provides a reliable scenario of what happens in our community. The insertion and removal of the implant was performed in a single center so that we were able to verify the actual time of continuity with the implant, avoiding self-report recall bias. A limitation of this study is the rate of losses in the first year, 14%, which corresponded to those women who moved to other Spanish regions, a situation caused by the COVID-19 pandemic in 2020. We do not believe that these losses have substantially modified the results, but we do not know for sure. Other limitations were the exclusion of women with low proficiency in Spanish and intellectual disability has meant that two very vulnerable groups of women are not part of this research. Future research would have to establish measures to avoid this bias. This study analyzed the variable place of birth, which dichotomized women into two groups: those born in Spain and those born in other countries but did not include the variable race or ethnic group, which could have shown differences in withdrawal behavior that could be related to cultural factors. Other variables not included in the study such as sexual orientation, gender identity or experience of gender violence or belonging to the gypsy ethnic group could have improved information on vulnerable situations that can modify women’s contraceptive behavior. Finally, the design of the study prevents us from exploring the psychological or social reasons that lead women to accept or not accept the side effects of the implant. Qualitative or mixed studies could shed light on women’s contraceptive expectations and needs and on clinicians’ biases in contraceptive counseling.

## Conclusion

This study shows that out of every 100 implants inserted, 17 will have been removed before one year, wasting an important part of the economic effort and health resources invested in their placement and increasing the risk of unwanted pregnancy. One factor that would significantly reduce the risk of early removal, and which is easily rectifiable, is to work toward ensuring that all women, before requesting an implant, make an appointment with the midwife at their health center for comprehensive and reliable information on the expected adverse effects.

### Limitation

This is the first planned interim analysis and includes 199 women. The whole three-year cohort will (three years) be present in future studies.

## Data availability statement

The raw data supporting the conclusions of this article will be made available by the authors, without undue reservation.

## Ethics statement

The studies involving humans were approved by committee of La Rioja (Ref. CEImLAR P.I 386). The studies were conducted in accordance with the local legislation and institutional requirements. The participants provided their written informed consent to participate in this study.

## Author contributions

RR: conceptualization, methodology, formal analysis, writing—original draft preparation, and writing—review and editing. RG-S: investigation, validation, and project administration. RU-M: investigation, validation, and writing—original draft preparation. PS-C: investigation, writing—original draft preparation, and project administration. AG-R: investigation and methodology. RJ-V: supervision. JS-G: software. AL-J: resources. ER-A: data curation. NN-E: writing—review and editing and funding. IS-A: supervision and writing—review and editing. All authors have read and agreed to the published version of the manuscript.
